# Improvement of Cancer Care—Analysis of ESGO Quality Indicators for the Surgical Treatment of Endometrial Cancer in Slovenian and Croatian Gynaecologic Oncology Departments

**DOI:** 10.3390/medicina61111901

**Published:** 2025-10-23

**Authors:** Maja Pakiž, Marko Klarić, Andraž Dovnik, Gabrijela Sopta Primorac, Jure Knez, Đuro Despot, Leyla Al Mahdawi, Marina Pranjic, Aleks Brumec, Tadej Turković, Andrej Cokan

**Affiliations:** 1 Department for Gynaecologic and Breast Oncology, Division for Gynaecology and Perinatology, University Medical Centre Maribor, 2000 Maribor, Slovenia; andraz.dovnik@ukc-mb.si (A.D.); jure.knez@ukc-mb.si (J.K.); almahdawi@gmail.com (L.A.M.); aleks.brumec@student.um.si (A.B.);; 2 Department for Obstetrics and Gynaecology, Clinical Hospital Centre Rijeka, 51000 Rijeka, Croatia; marko.klaric2@medri.uniri.hr (M.K.); gsopta@gmail.com (G.S.P.); drdurodespot@gmail.com (Đ.D.); pranjicmarina10@gmail.com (M.P.); matejturkovic@gmail.com (T.T.)

**Keywords:** quality indicators, improvement of cancer care, endometrial cancer

## Abstract

*Background and Objectives:* This study aims to analyze ESGO quality indicators for the surgical treatment of endometrial cancer patients at two gynecologic oncology departments in Slovenia and Croatia, providing insights for improving cancer care. *Materials and Methods:* We conducted a retrospective analysis of clinical data to evaluate ESGO quality indicators for surgical treatment of endometrial cancer patients from 2020 to 2022. ESGO quality indicators were calculated, and the results were discussed to formulate suggestions for enhancing cancer care. Institutional review board approval was obtained for the analysis. *Results:* The analysis reveals that the Slovenian Department for Gynecology and Breast Oncology in Maribor achieved compliance with 24 out of 26 quality indicators, while the Department of Obstetrics and Gynecology in Rijeka met 18 out of 26. The disparity may be partly attributed to Maribor’s status as an ESGO-accredited center in training since 2014, which facilitates more rapid updates in care practices. *Conclusions:* Based on our findings and epidemiological data, we recommend several actions to enhance cancer care in both countries: (a) advance initiatives for the centralization of care (2 to 3 centers in Slovenia and 5 to 6 in Croatia), (b) implement a national system for the prospective measurement of quality indicators, and (c) pursue center accreditation and gynecologist certification in subspecialty care by international societies such as ESGO, given the small size of both countries. Our results confirm that the analysis of ESGO quality indicators is a viable method for all stakeholders involved in enhancing cancer care.

## 1. Introduction

Improving the quality of cancer care is a key mission across all levels of healthcare, from individual centers to national and international societies, policymakers, and EU-level programs. It is estimated that 30–40% of patients in the general population receive treatment that is not evidence-based, with 20–25% receiving unnecessary or potentially harmful interventions [1]. Establishing quality care begins with developing transparent, evidence-based guidelines, followed by creating quality indicators to assess guideline adherence through clinical audits. Ideally, meeting these indicators would be linked to center accreditation and physician certification. In gynecologic oncology, the European Society of Gynaecological Oncology (ESGO) plays a pivotal role in this process [2,3,4,5,6].

Endometrial cancer, the most common gynecologic malignancy, is increasing in incidence in developed countries owing to lifestyle factors (e.g., obesity and diabetes) and demographic shifts [7,8]. Accordingly, policymakers and public health organizations need high-quality data and tools to develop networks of centers that deliver optimal care for patients with endometrial cancer. Several publications have clearly described the importance of comprehensive management of cancer patients in tertiary-level or teaching hospitals in general, in the population with gynecologic cancers, and specifically in the population of patients with endometrial cancer. The importance of centralization of care and treatment by specialized gynecologists is reflected in better survival outcomes, faster adoption of novel surgical approaches, and better adherence to updated international guidelines [9,10,11,12,13,14]. Already in 2014, a clear need was identified for measuring adherence to guidelines in quality-assurance programs in gynecologic oncology [15]. Scientific data clearly recommend centralization of care; however, data on how many endometrial cancer patients are treated outside tertiary-level or teaching hospitals are scarce. Within Europe, the number of published articles is minimal; for instance, reports from Germany and Poland suggest that most EC patients are actually treated at general or secondary-level hospitals. [16,17]. Denmark represents a notable—and exceptionally rare—success in the centralization of gynecologic cancer care [18]. Developing networks of centers of clinical excellence for gynecologic oncology—and specifically for endometrial cancer—remains a major gap in most countries, potentially leading to inequitable access to guideline-concordant management, as recently emphasized in ESGO’s contribution to the EU public consultation [19].

Clinical quality indicators are an essential part of measuring the quality of health care. These indicators are divided into three groups that measure health care structure, processes, and outcomes. To ensure that quality indicators are useful and reliable tools for measuring and improving health care quality, they must be designed, defined, and implemented with scientific rigor. Quality indicators should be valid for the majority of patients with a specific disease [20]. The use of quality indicators and feedback has a small-to-moderate impact on individual professional performance [21]; however, they may also be used for institutional quality-assurance programs, governmental quality assessment, and as tools for accreditation processes [3].

Targeting process quality indicators, or combining them with outcome indicators, seems to be the most efficient feedback tool for improving care [22]. Measurement of quality indicators can therefore enhance implementation of new clinical guidelines in everyday practice. Implementation of guidelines is a key step toward improving patient care and avoiding unnecessary or even harmful care; however, it is associated with multiple barriers, including personal factors, guideline-related factors, and external factors [1]. Implementation science has been developed to increase the use of theoretical approaches to better understand and explain how and why implementation succeeds or fails. Defining barriers and enablers for successful implementation of guidelines, and identifying the best model for implementation programs, is an important step for all stakeholders [23,24]. The gap in successful implementation of guidelines may therefore be partly closed by the systematic use of clinical quality indicators by the different stakeholders involved in healthcare policy.

ESGO, together with ESTRO and ESP, developed comprehensive guidelines for endometrial cancer treatment [2]. The guidelines were updated in July 2025 [4], further emphasizing the FIGO 2023 staging system [25] and the systematic use of molecular genetic testing in all endometrial cancer patients [4]. The guidelines are evidence-based, openly accessible, and were developed, among other methods, with external review to assess implementability across regions and institutions. To accelerate guideline implementation and support quality assurance, ESGO also established quality indicators (QIs) for the surgical management of endometrial cancer, which are used for ESGO accreditation of centers [3,6]. The ESGO QIs were developed by identifying scientific experts, performing systematic literature research by specialized methodologist, designing specific indicators and evaluating them by a large panel of practicing clinicians. The process followed principles of implementation science [3].

This study analyzes compliance with ESGO quality indicators (QIs) for endometrial cancer surgery in two university-level gynecologic oncology centers—one in Slovenia and one in Croatia. The centers are comparable in size, case volume, and historical background. The primary objective was to assess QI fulfillment and compare performance in a narrative manner between the two centers, providing insights for improvement through clinical audit. A secondary aim was to raise awareness of the relevance of prospective evaluation of standardized, universal QIs to help close gaps in guideline implementation and insufficient centralization of care, which can lead to inequitable access to high-quality care for patients with endometrial cancer.

The findings of the study are intended to encourage quality improvement and should not be used punitively against physicians or institutions.

## 2. Methods

### 2.1. The Study Design and Setting

The retrospective clinical audit-based study was conducted at two gynecologic oncology centers: the Department of Gynecologic and Breast Oncology, University Medical Centre Maribor, Slovenia (MB center), and the Department of Obstetrics and Gynecology, Clinical Hospital Centre Rijeka, Croatia (RI center). MB center was ESGO-accredited in training in 2014, while RI center, though not yet accredited, is highly motivated to apply for ESGO accreditation.

### 2.2. Study Endpoints

The primary endpoint was the percentage of QIs meeting their target in each center. Secondary endpoints included measuring all QIs, comparing QI results between centers in a narrative way, assessing data availability, and formulating recommendations for future organizational improvements.

### 2.3. Obtaining the Data and Study Population

All QIs were intended to be assessed. Both centers completed the standard ESGO accreditation table (in Excel) provided by ESGO to ensure consistency; the Excell sheet defines the specific parameters subsequently used to calculate or describe the published QIs [3]. At both centers, data for all consecutive endometrial cancer patients treated from 2020 to 2022 (the study population for QI calculation) were extracted from hospital records and department databases. Data from all consecutive endometrial cancer patients were included; no patients were excluded. The original ESGO Excell sheet and the parameters collected did not allow for direct calculations of three QI (for QI 14, QI 15, and QI 18), they were assessed in a narrative way. The obstacles were that the dataset did not require recording whether SNB was attempted (QI15); only whether SNB or lymphadenectomy (LND) was performed was captured. Consequently, the rate of ipsilateral LND when the sentinel node was not detected could not be calculated (“LND performed” denoted either upfront LND or LND due to failed SN detection) (QI18). For QI14, only final pathology reports were collected; preoperative data were not captured. Furthermore, some MB patients were enrolled in the TESLA-1 trial, which required LND after SNB. A summary of the QIs is shown in Table 1. The results are presented according to the numbering and calculations of ESGO QIs as outlined in the referenced publication and described in the methods [3].

A personal interview at RI center was conducted by the first and second author to gather additional information on some process and outcome QIs (non-numerical) and to perform cross-checking.

### 2.4. Analysis of Results

The analysis was narrative, as not all QIs are numerical; therefore, statistical comparisons of QIs between centers were not feasible. The aim of the study was to assess fulfillment of published QIs [3] by each center separately and not to compare specific clinical outcomes between the centers.

### 2.5. Ethical Considerations

The study was approved by the institutional review boards of both centers (RI 2170-29-02/1-24-2, MB UKC-MB-KME-66/25), and informed consent was waived due to the retrospective, anonymized nature of the data.

## 3. Results

Between 2020 and 2022, the two centers managed 314 newly diagnosed endometrial cancer patients: 183 at MB and 131 at RI (QI1). At both centers, >90% of new cases and all recurrences were discussed at a multidisciplinary team meeting before treatment (QI4). The few not presented preoperatively involved patients initially operated for presumed benign disease, with cancer discovered incidentally.

In MB, patients participated in two prospective trials—TESLA-1 and EUGENIE. In 2020, MB also initiated the process of joining an ENGOT/CEEGOG phase II/III industry-sponsored trial (navtemadlin maintenance therapy for TP53-wild-type advanced or recurrent endometrial cancer). No prospective trials were ongoing at RI (QI5).

More than 90% of patients at both centers underwent preoperative work-up in accordance with ESGO/ESTRO/ESP guidelines, except for those initially treated for presumed benign conditions in whom endometrial cancer was diagnosed incidentally (QI6).

Both centers reported that >99% of patients had the uterus removed intact, without morcellation or intraoperative rupture; exceptions involved cases initially operated for presumed benign disease (QI8).

At MB, two intraoperative bowel injuries and one minor bladder injury occurred (3/183; 1.6%), all related to dense adhesions; one case required reoperation. No major intraoperative injuries were recorded at RI over the three-year period (QI12).

In both centers, lymph node assessment was performed in 99% of presumed early-stage cases. In MB, nearly all SNBs (99/101; 99%) were performed by gynecologic oncologists (*n* = 4); two cases were undertaken by a new fellow under supervision. The mean number of SNBs per surgeon per year was 13.5 (QI16). In RI, gynecologic oncologists (*n* = 5) performed 86.8% (59/68) of SNBs, with a mean of 6.3 per surgeon per year (QI16).

All identified sentinel lymph nodes underwent ultrastaging at both centers (QI19). At MB, all patients received molecular classification in accordance with ESGO/ESTRO/ESP guidelines; p53 and MMR were performed in-house, and POLE testing was outsourced via an established pathway. At RI, p53 and MMR were assessed, but POLE testing was not performed as not being available (QI23).

Post-surgery, all patients in both centers were discussed in multidisciplinary team meetings, with adjuvant treatment aligned with ESGO/ESTRO/ESP guidelines. The ESGO application aids clinicians in decision-making and was used regularly at both centers (QI24). In MB, surgical reports are structured and uniform across all surgeons, including the items listed in QI25; in RI, reports are not structured.

In both centers, pathology reports include all items required by QI26, and structured reporting was implemented during the study period. Multidisciplinary morbidity and mortality (MM) conferences are held in cases of major complications or unexpected postoperative deaths, as mandated by hospital management, with a uniform structured report. All patients are discussed before and after surgery and in recurrent/metastatic settings; however, complications are not reported in a structured format (QI27).

In MB, a prospective database tracks all new cases, including complications and deaths. Hospital management systematically reports deaths, perioperative complications, and thromboembolic events to the department chief (QI29). Reoperations within 30 days after minimally invasive surgery were rare—one case (0.5%) at MB due to a bowel injury. RI reported no reoperations within 30 days over the three-year period (QI28).

Both centers automatically report all recurrences and deaths to the national cancer registry, as required by law. Survival data are managed centrally by the national registries, not by the centers themselves (QI29). All other QIs are presented in Table 2.

## 4. Discussion

The study confirmed that the MB center, ESGO-accredited since 2014, achieved a higher proportion of QI targets than the RI center (24/26 [92.3%] vs. 18/26 [69.2%]). In both centers, three QIs (QI14, QI15, QI18) were not directly assessed due to lack of data in a calculable format and were evaluated narratively. At MB, assessment of QI14, QI15 and QI18 was further limited by enrollment of patients in the TESLA-1 trial [26].

ESGO QIs for the surgical treatment of endometrial cancer are categorized into structural, process, and outcome indicators.

### 4.1. Structural QIs (Table 1; QI1, QI2, QI5)

MB met all three structural QIs, although its volume was at the minimum required threshold. Cases diagnosed at the end of December or the beginning of January were assigned to either the preceding or the subsequent year, but not both. RI was slightly below the targets for QI1 and QI2 and did not meet QI5, as no prospective trials were conducted.

Slovenia (2 million inhabitants) and Croatia (4 million) share a similar healthcare history as former Yugoslav republics. Both have solidarity-based systems, with public providers delivering most care. Neither country has a formal, government-led policy for centralization of cancer care. In Croatia, all gynecologic departments may treat cancer patients, although the Ministry of Health designates “reference centers” for second opinions in gynecologic oncology. In Slovenia, all gynecologic departments can treat endometrial cancer; centralization has been mildly encouraged by some governmental bodies, but no formal implementation strategy exists.

Therefore, because all hospitals treating endometrial cancer in these two countries are state-run, the lack of action by the respective Ministries of Health to mandate centralization is the principal reason it has not been implemented.

Epidemiological data from national cancer registries report approximately 370 new endometrial cancer cases annually in Slovenia [27] and 750 in Croatia [28]. To optimize care in line with QI targets, an ideal configuration would be 2–3 centers in Slovenia and 5–6 in Croatia, and to ensure that no patients are treated outside specialized centers. Development of a network of centers of clinical excellence for endometrial cancer—and gynecologic cancers more broadly—based on scientific evidence is an ESGO priority, as reflected in the ESGO accreditation program [6]. ESGO has initiated EU-level advocacy to raise awareness and promote an evenly distributed network of centers of excellence to ensure equal and timely access to high-quality care for all EU citizens [19]. These activities aim to prompt national and local policymakers to undertake country-specific restructuring. In Europe, Denmark provides an excellent example of successful, Ministry-led centralization of care [18,29].

Small-volume centers often struggle to conduct prospective research owing to limited patient numbers. Joining international research groups can expand access to trials, although bureaucratic requirements and language differences pose substantial barriers. Our results and interviews suggest that ESGO accreditation increases awareness of, and participation in, international research networks such as Central and Eastern European Gynecologic Oncology Group (CEEGOG), of which MB is a member. This affiliation has enabled MB to host more prospective trials than the RI center.

### 4.2. Process QIs (Table 1; QI 3, QI 4, QI 23, QI 25, QI 26)

All listed QIs are met in MB. In RI, QI 4 (multidisciplinary tumor board discussions) and QI 26 (pathologic report requirements) are fully met; however, about 25% of patients are treated by general gynecologists (QI 3), the surgical report is unstructured (QI 25), and POLE is unavailable (QI 23).

In Croatia, subspecialty training in gynecologic oncology is established but not mandatory for treating gynecologic cancer patients. Slovenia lacks a formal gynecologic oncology subspecialty program; only one clinician has completed ESGO subspecialty training [5] and is employed at the MB center. The MB center’s organization, with a dedicated gynecologic oncology department, facilitates subspecialization through practice and experience aligned with the ESGO curriculum. All gynecologists at MB devote over 80% of their time to gynecologic patients. Both countries could benefit from making international subspecialty training and licensing mandatory for clinicians treating gynecologic cancer, as evidence suggests that a surgeon’s subspecialty background positively impacts oncologic outcomes [9].

Our results indicate that both centers recognize the importance of multidisciplinary treatment planning and have integrated it into daily practice. Decisions made during multidisciplinary team meetings are more likely to align with evidence-based guidelines than those made by individual physicians [30,31,32]. Centralization of care enables access to multiple specialists and necessary equipment in one location, ensuring sustainable use of resources and efficient clinical pathways. As both centers are part of university hospitals, they benefit from the availability of specialists and organized multidisciplinary team meetings, further supporting the need for centralization in both countries.

Molecular markers for endometrial cancer are rapidly emerging diagnostic tools that significantly influence treatment decisions [2,3,33]. POLE is a molecular genetic marker that strongly informs adjuvant treatment decisions; however, its use requires specialized laboratory equipment and expertise and entails additional direct costs [34]. Consequently, it may not be available or reimbursed. Our ESGO QI–based analysis can help departments advocate to hospital management and health insurers for access to POLE testing, whether in-house or via referral.

### 4.3. Outcome ESGO QI (Table 1; QI 6–22, QI 24, QI 27, QI 28, QI 29)

Both centers have implemented modern management for endometrial cancer; however, some QIs do not meet target values. The RI center has not yet fully adopted the requirement that subspecialists perform all surgeries, contributing to a low annual number of sentinel node biopsies (SNBs) per surgeon and failure to meet the bilateral mapping target. Conversely, the MB center achieved the bilateral mapping target but did not reach the recommended number of SNBs per surgeon (Table 2).

Operating in a small country yields smaller centers, even at the university level. Centers must balance new patients per surgeon with continuous staffing, accounting for leave, education, and illness. The MB center appears to have an optimal number of surgeons, whereas the RI center may benefit from one fewer. At RI, the gynecology and obstetrics department is not divided into subspecialty units (as at MB); consequently, non–gynecologic oncology duties are harder to buffer, limiting surgeons’ dedicated time to gynecologic oncology and reducing procedures per surgeon. Both centers mitigate lower per-surgeon volume by assigning two dedicated surgeons to most operations. Further centralization and the creation of subspecialty units dedicated to gynecologic cancer within a limited number of centers would increase case volumes and enhance care quality.

Our analysis revealed that although both centers possess all necessary data, it is not organized to allow automatic QI calculation. Consequently, QI14, QI15, and QI18 were assessed narratively and calculated manually. At the MB center, evaluation of these QIs was further constrained by enrollment in the TESLA-1 trial, which required a specific lymph-node staging protocol.

These indicators pertain to the assessment of the sentinel node biopsy (SNB) algorithms used by the centers. According to current guidelines, surgical lymph-node assessment is not required for low-risk endometrial cancer, although SNB may be performed. For intermediate-high- and high-risk patients, surgical lymph-node assessment is recommended, and SNB is an acceptable method [2,4]. If the sentinel node is not identified, a site-specific lymphadenectomy should be performed.

Both centers use the Memorial Sloan Kettering Cancer Center algorithm [35]; accordingly, we consider QIs 14, 15, and 18 to be probably met.

Prospective collection and analysis of perioperative complications, with appropriate documentation, are essential for quality control. Currently, neither country has a national quality-control program that systematically captures indicators such as complications, readmissions, and reoperations. Both departments monitor patients and maintain prospective databases, and they follow hospital guidance for documenting multidisciplinary team meetings. However, implementing an objective tool for grading and analyzing perioperative complications—such as the Clavien–Dindo classification [36]—is recommended for both centers. Similarly, although there is substantial evidence that ERAS improves perioperative outcomes and quality of life in endometrial cancer patients [37], implementation remains suboptimal [38,39]. Prospective clinical audit, together with measurement of guideline adherence, may enhance ERAS implementation. As with centralization of care, coordinated efforts from all stakeholders—both management leadership and bottom-up engagement—and continuous programs to sustain guideline implementation are required to achieve durable improvements in healthcare. Illustrative programs can be observed within the Alberta Health Services in Canada [40,41].

Both centers recognize that the published ESGO QIs are valuable for performance assessment and serve as practical guides for future organizational changes. These indicators provide an objective tool for engaging with hospital management and national policy bodies. In both countries, national cancer registries are well established and capture all cancer patients [27,28]. However, they do not routinely collect quality-control indicators. Denmark’s cancer registry provides an excellent example of integrating quality indicators into a national registry, offering a robust tool to ensure high—and comparable—quality across centers delivering cancer care [29]. Future studies—primarily in implementation science—will identify the most effective and efficient strategies and models to improve guideline implementation and the centralization of care.

#### Strengths and Limitation

The strengths of this study are the systematic, consistent evaluation of quality indicators (QIs) in two comparable centers, demonstrating that these indicators are user-friendly and generalizable to all centers treating patients with endometrial cancer. The limitations are that some process and outcome QIs were assessed through personal interviews and site visits among the authors and are therefore partially based on trust.

## 5. Conclusions

According to our result, included ESGO-accredited center showed higher compliance with ESGO quality indicators for endometrial cancer treatment compared with a similarly positioned center in the healthcare system with no accreditation. Prospective collection and analysis of quality indicators enable objective self-assessment, inform organizational planning, and support national policies aimed at improving cancer care. This analysis confirms the feasibility of using ESGO quality indicators as a practical method for stakeholders to enhance care. Furthermore, establishing national and EU-wide prospective databases with standardized data collection enabling automatic quality-indicator calculation would be a valuable tool to support guideline implementation, harmonize cancer care, and reduce disparities in access to high-quality treatment.

## Figures and Tables

**Table 1:** Overview of ESGO quality indicators. QI-quality indicator, MIS-minimally invasive surgery, BMI-body mass index, SNB-sentinel node biopsy.

Structural ESGO QI	Process ESGO QI	Outcome ESGO QI
(a) Number of newly diagnosed cases of endometrial carcinoma treated per centre per year (QI1). (b) Number of endometrial carcinoma primary surgeries performed per year (QI2). (c) Centre participating in ongoing prospective studies (QI5).	(a) Surgery performed by subspecialized/dedicated gynaecologic surgeon (QI3). (b) Treatment plan discussed at multidisciplinary team meeting (QI4). (c) Proportion of patients undergoing complete molecular classification (QI23). (d) Minimum required elements in surgical reports (QI25). (e) Minimum required elements in pathology report (QI26).	(a) Proportion of patients with pre-operative work-up according to ESGO/ESTRO/ESP guidelines (QI6). (b) Proportion of presumed FIGO stage I-II upstaged to IVB (QI7). (c) Proportion of patients with non-ruptured uterus after hysterectomy (QI8). (d) Proportion of patients who undergone successful MIS (QI9). (e) Proportion of patients with BMI > 35 who undergone successful MIS (QI10). (f) Proportion of conversions from MIS to laparotomy (QI11). (g) Proportion of patients with intra-operative injuries (QI12). (h) Proportion of infracolic omentectomy in presumed early stage serous, undifferentiated carcinoma or carcinosarcoma (QI13). (i) Proportion of lymph node staging in high-intermediate, high-risk patients (QI14). (j) Proportion of SNB (QI15). (k) Number of SNB per surgeon per year (QI16). (l) Proportion of ICG in SNB (QI17). (m) Proportion of patients with site-specific LND if no SN mapping (QI18). (n) Proportion of ultrastaging of SN (QI19). (o) Proportion of bilateral mapping of SN (QI20). (p) Proportion of macroscopic complete resection when curative intention for advanced stage (QI21). (q) Proportion of macroscopic complete resection of recurrent disease in salvage surgery (QI22). (r) Compliance with ESGO/ESTRO/ESP adjuvant treatment guidelines (QI24). (s) Structured morbidity and mortality conference per year (QI27). (t) Proportion of reoperations within 30 days (QI28). (u) Structured prospective reporting of recurrences/deaths (QI29).

**Table 2:** Results of selected quality indicator.

	MB Centre (ESGO Accredited in Training)	RI Centre	Target	Comment
All new endometrial cancer patients (three years period) (QI 1)	183	131	Optimal ≥ 90 per year Minimal: ≥50 per year	
No. of surgically treated patients (three years period) (QI 2)	150	122	Optimal ≥ 80 Minimal: ≥50	
No. of patients treated by dedicated gynaecologic oncologist (QI 3)	144/150 (96.0%)	91/122 (74.6%)	≥95%	
Upgrade of presumed early-stage disease to FIGO IV (QI 7)	2/135 (1.5%)	0	<5%	
Conversion to laparotomy (QI 11)	6/166 (5.2%)	4/72 (5.6%)	<10%	
Successful MIS (QI 9)	110/135 (81%)	68/106 (64.2%)	Optimal: ≥80% Minimal: 60%	
Successful MIS (QI 10)	26/31 (83.8%)	11/17 (64.7%)	>60%	
Complete resection of advanced/recurrent disease (QI 21)	12/14 primary advanced (85.7%)	5/10 primary advanced (50%) 4/6 recurrent (66.6%)	≥75%	
Staging omentectomy (QI 13)	2/9 (22%)	5/18 (27.8%)	≥90%	Only data about final pathology are available
Using ICG for SNB (QI 17)	98/101 (97.0%);	68/68 (100%)	≥95%	Radioactive tracer used alone when technical issues with ICG equipment. Radioactive tracer used in patients enrolled in TESLA-1 study.
Bilateral detection rate (QI 29)	80/101 (79.2%)	44/68 (64.7%)	≥75%	
Pelvic LND if unilateral detection/no mapping (QI 18)	Can not be assessed due to the prospective trial	13/13 (100%)	>90%	In MB endometrial cancer some patients were being enrolled in TESLA-1 study (which necessitate bilateral LND after SNB)
Presumed early-stage disease and non endometioid G1/2 disease (QI 14, QI 15)	31/31 lymph node status was done; 100% SNB was attempted 16/31 (51.6%) SNB 23/31 (74.2%) LND	19/19 lymph nodes status done; 100% SNB was attempted,12/19 (63.2%) SNB 8/19 (42.1%) LND	>85% (QI 14) 90% (QI 15)	LND performed when failed detection of SN or if being enrolled in TESLA-1 (in MB)

## Data Availability

The original contributions presented in this study are included in the article. Further inquiries can be directed to the corresponding author.

## References

[1] Fischer F., Lange K., Klose K., Greiner W., Kraemer A. (2016). Barriers and strategies in guideline implementation: A scoping review. Healthcare.

[2] Concin N., Matias-Guiu X., Vergote I., Cibula D., Mirza M.R., Marnitz S., Ledermann J., Bosse T., Chargari C., Fagotti A. (2021). ESGO/ESTRO/ESP guidelines for the management of patients with endometrial carcinoma. Int. J. Gynecol. Cancer.

[3] Concin N., Planchamp F., Abu-Rustum N.R., Ataseven B., Cibula D., Fagotti A., Fotopoulou C., Knapp P., Marth C., Morice P. (2021). European Society of Gynaecological Oncology quality indicators for the surgical treatment of endometrial carcinoma. Int. J. Gynecol. Cancer.

[4] Concin N., Matias-Guiu X., Cibula D., Colombo N., Creutzberg C.L., Ledermann J., Mirza M.R., Vergote I., Abu-Rustum N.R., Bosse T. (2025). ESGO–ESTRO–ESP guidelines for the management of patients with endometrial carcinoma. Lancet Oncol..

[5] European Society of Gynaecological Oncology (ESGO) Fellowship Training. https://esgo.org/fellowship-training.

[6] European Society of Gynaecological Oncology (ESGO) Centre Accreditations. https://esgo.org/explore/esgo-accreditation.

[7] European Commission Joint Research Centre (JRC) ECIS—European Cancer Information System. https://ecis.jrc.ec.europa.eu/.

[8] Baker-Rand H., Kitson S.J. (2024). Recent advances in endometrial cancer prevention, early diagnosis and treatment. Cancers.

[9] Chan J.K., Sherman A.E., Kapp D.S., Zhang R., Osann K.E., Maxwell L., Chen L.M., Deshmukh H. (2011). Influence of gynecologic oncologists on the survival of patients with endometrial cancer. J. Clin. Oncol..

[10] Wright J.D., Burke W.M., Tergas A.I., Hou J.Y., Huang Y., Hu J.C., Hillyer G.C., Ananth C.V., Neuget A.I., Hershman D.L. (2016). Comparative effectiveness of minimally invasive hysterectomy for endometrial cancer. J. Clin. Oncol..

[11] Lam M.B., Riley K.E., Mehtsun W., Phelan J., Orav E.J., Jha A.K., Burke L.G. (2021). Association of teaching status and mortality after cancer surgery. Ann. Surg. Open.

[12] Rodriguez V.E., LeBrón A.M., Chang J., Bristow R.E. (2021). Guideline-adherent treatment, sociodemographic disparities, and cause-specific survival for endometrial carcinomas. Cancer.

[13] Wollinga T., Ezendam N.P., Eggink F.A., Smink M., van Hamont D., Pijlman B., Boss E., Robbe E.J., Ngo H., Boll D. (2018). Implementation of laparoscopic hysterectomy for endometrial cancer over the past decade. Gynecol. Surg..

[14] Buskwofie A., Huang Y., Tergas A.I., Hou J.Y., Ananth C.V., Neugut A.I., Hershman D.L., Wright J.D. (2018). Impact of hospital volume on racial disparities and outcomes for endometrial cancer. Gynecol. Oncol..

[15] Ferron G., Martinez A., Gladieff L., Mery E., David I., Delannes M., Montastruc M., Balagué G., Picaud L., Querleu D. (2014). Adherence to guidelines in gynecologic cancer surgery. Int. J. Gynecol. Cancer.

[16] Hansinger J., Völkel V., Gerken M., Schoffer O., Wimberger P., Bierbaum V., Bobeth C., Rößler M., Dröge P., Ruhnke T. (2024). Endometrial Cancer—Long-Term Survival in Certified Cancer Centers and Non-Certified Hospitals: Comparative Analysis Based on a Large German Retrospective Cohort Study (WiZen). Geburtshilfe Frauenheilkd..

[17] Piątek S., Urbański F., Karczmarz S., Prusaczyk A., Sobiczewski P., Bogdan M., Gujski M., Bidziński M. (2023). Assessment of hospital volume in the surgical management of endometrial and ovarian cancer: A Polish population-based study. Med. Sci. Monit..

[18] Jørgensen S.L., Mogensen O., Wu C., Lund K., Iachina M., Korsholm M., Jensen P.T. (2019). Nationwide introduction of minimally invasive robotic surgery for early-stage endometrial cancer and its association with severe complications. JAMA Surg..

[19] European Society of Gynaecological Oncology (ESGO) Advocacy: EU Public Consultation. https://www.esgo.org/advocacy/.

[20] Mainz J. (2003). Defining and classifying clinical indicators for quality improvement. Int. J. Qual. Health Care.

[21] Cochrane Audit and Feedback: Effects on Professional Practice. https://www.cochrane.org/evidence/CD000259_audit-and-feedback-effects-professional-practice.

[22] Ali M.P., Visser E.H., West R.L., van Noord D., van der Woude C.J., van Deen W.K. (2025). Reporting feedback on healthcare outcomes to improve quality in care: A scoping review. Implement. Sci..

[23] Nilsen P. (2015). Making sense of implementation theories, models and frameworks. Implement. Sci..

[24] De Mello N.F., Silva S.N., Gomes D.F., Girardi J.D.M., Barreto J.O.M. (2024). Models and frameworks for assessing the implementation of clinical practice guidelines: A systematic review. Implement. Sci..

[25] Ferrari F., Gozzini E., Conforti J., Giannini A., Barra F., Fichera A., Ferrari F.A., Majd H.S., Odicino F. (2025). Impact of the FIGO 2023 staging system on the adjuvant treatment of endometrial cancer: A comparative analysis with FIGO 2009. Cancers.

[26] Pakiz M., Cibula D., Wydra D.G., Klat J., Zikan M., Matylevich O., Poncova R., Abacjew-Chmylko A., Cokan A., Romanova M. (2025). Sentinel node biopsy using two concurrent labeling techniques (radioactive tracer with/without blue dye vs. Indocyanin Green-ICG) in early-stage endometrial cancer patients (TESLA–1): A prospective observational study CEEGOG EX-02. Cancers.

[27] Institute of Oncology Ljubljana (2020). Rak v Sloveniji—Cancer in Slovenia 2020.

[28] Croatian Institute of Public Health (HZJZ) (2024). Incidencija Raka u Hrvatskoj u 2021. Godini.

[29] Sørensen S.M., Bjørn S.F., Jochumsen K.M., Jensen P.T., Thranov I.R., Hare-Bruun H., Seibæk L., Høgdall C. (2016). Danish Gynecological Cancer Database. Clin. Epidemiol..

[30] Castel P., Tassy L., Lurkin A., Blay J.-Y., Meeus P., Mignotte H., Faure C., Ranchere-Vince D., Bachelot T., Guastalla J.-P. (2012). Multidisciplinarity and medical decision, impact for patients with cancer: Sociological assessment of two tumour committees’ organization. Bull. Cancer.

[31] Evans A.C., Zorbas H.M., A Keaney M., A Sidhom M., E Goodwin H., Peterson J.C. (2008). Medicolegal implications of a multidisciplinary approach to cancer care: Consensus recommendations from a national workshop. Med. J. Aust..

[32] Crawford R., Greenberg D. (2012). Improvements in survival of gynaecological cancer in the Anglia region of England: Are these an effect of centralisation of care and use of multidisciplinary management?. BJOG Int. J. Obstet. Gynaecol..

[33] León-Castillo A., Gilvazquez E., Nout R., Smit V.T., McAlpine J.N., McConechy M., Kommoss S., Brucker S.Y., Carlson J.W., Epstein E. (2020). Clinicopathological and molecular characterisation of ‘multiple-classifier’ endometrial carcinomas. J. Pathol..

[34] Plotkin A., Olkhov-Mitsel E., Nofech-Mozes S., Djordjevic B., Mirkovic J., Fitzpatrick M., Krizova A., Hong N.J.L. (2023). Budget impact analysis of molecular subtype profiling in endometrial cancer. Gynecol. Oncol..

[35] Barlin J.N., Khoury-Collado F., Kim C.H., Leitao M.M., Chi D.S., Sonoda Y., Alektiar K., DeLair D.F., Barakat R.R., Abu-Rustum N.R. (2012). The importance of applying a sentinel lymph node mapping algorithm in endometrial cancer staging: Beyond removal of blue nodes. Gynecol. Oncol..

[36] Clavien P.A., Barkun J., de Oliveira M.L., Vauthey J.N., Dindo D., Schulick R.D., de Santibañes E., Pekolj J., Slankamenac K., Bassi C. (2009). The Clavien–Dindo classification of surgical complications: Five-year experience. Ann. Surg..

[37] Ferrari F., Majd H.S., Giannini A., Favilli A., Laganà A.S., Gozzini E., Odicino F. (2024). Health-related quality of life after hysterectomy for endometrial cancer: The impact of enhanced recovery after surgery shifting paradigm. Gynecol. Obstet. Investig..

[38] Bhandoria G.P., Bhandarkar P., Ahuja V., Maheshwari A., Sekhon R.K., Gultekin M., Ayhan A., Demirkiran F., Kahramanoglu I., Wan Y.-L.L. (2020). Enhanced Recovery After Surgery (ERAS) in gynecologic oncology: An international survey of peri-operative practice. Int. J. Gynecol. Cancer.

[39] Gómez-Hidalgo N.R., Pletnev A., Razumova Z., Bizzarri N., Selcuk I., Theofanakis C., Zalewski K., Nikolova T., Lanner M., Kacperczyk-Bartnik J. (2023). European Enhanced Recovery After Surgery (ERAS) gynecologic oncology survey: Status of ERAS protocol implementation across Europe. Int. J. Gynaecol. Obstet..

[40] Gramlich L.M., Sheppard C.E., Wasylak T., Gilmour L.E., Ljungqvist O., Basualdo-Hammond C., Nelson G. (2017). Implementation of Enhanced Recovery After Surgery: A strategy to transform surgical care across a health system. Implement. Sci..

[41] Gramlich L., Nelson G., Nelson A., Lagendyk L., Gilmour L.E., Wasylak T. (2020). Moving enhanced recovery after surgery from implementation to sustainability across a health system: A qualitative assessment of leadership perspectives. BMC Health Serv. Res..

